# Accuracy Improvement of Vehicle Recognition by Using Smart Device Sensors †

**DOI:** 10.3390/s22124397

**Published:** 2022-06-10

**Authors:** Tanmoy Sarkar Pias, David Eisenberg, Jorge Fresneda Fernandez

**Affiliations:** 1 Department of Computer Science, Virginia Tech, Blacksburg, VA 24061, USA; tanmoysarkar@vt.edu; 2 Department of Information Systems, Ying Wu College of Computing, New Jersey Institute of Technology, Newark, NJ 07102, USA; 3 Martin Tuchman School of Management, New Jersey Institute of Technology, Newark, NJ 07102, USA; fresneda@njit.edu

**Keywords:** vehicle recognition, CNN, signal processing, sensor, deep learning

## Abstract

This paper explores the utilization of smart device sensors for the purpose of vehicle recognition. Currently a ubiquitous aspect of people’s lives, smart devices can conveniently record details about walking, biking, jogging, and stepping, including physiological data, via often built-in phone activity recognition processes. This paper examines research on intelligent transportation systems to uncover how smart device sensor data may be used for vehicle recognition research, and fit within its growing body of literature. Here, we use the accelerometer and gyroscope, which can be commonly found in a smart phone, to detect the class of a vehicle. We collected data from cars, buses, trains, and bikes using a smartphone, and we designed a 1D CNN model leveraging the residual connection for vehicle recognition. The model achieved more than 98% accuracy in prediction. Moreover, we also provide future research directions based on our study.

## 1. Introduction

Handheld mobile devices have been an indispensable part of the global market since 1979 [1], and in recent years, have been expanding from phones to include watches and wristbands [2]. Consumers can now access location-based services (LBS) and mobile social networks (MSNs) through a mobile smart device that can track their location using cell towers, wireless fidelity (Wi-Fi), global positioning system (GPS), Bluetooth, and radio frequency identification (RFID), among other technologies. An accelerometer sensor (AS) and a gyroscope sensor (GS) are commonly used in mobile phones [3]. These sensors are especially functional for measuring three-dimensional object orientation or stationing, as well as changes in the ambient environment, as they have the ability to supply raw data with great clarity and accuracy.

Automatic wireless monitoring of motor vehicles saves many lives by preventing collisions, notifying drivers as well as ambulatory and emergency services when accidents take place. With GPS, apps for testing cars, ships, and trains have been built to help avoid or monitor problems [2].When applied to vehicle recognition, activity recognition technology can collect and assess a plethora of vehicular, driver, transit, and environmental data, from identifying any small bump in the road, to increasing the accuracy of vehicle recognition, and a range of driver and driving conditions. This information can be harnessed from the smart device, and wirelessly processed to wide-scale databases for the mining of market intelligence, in addition to direct customer support. Potholes may be identified even before they became major problems, when an Apple watch sends wireless movement data. Slick or unsafe driving conditions could be communicated in real time, after vehicles have passed over a specific point in the road. Gadgets can submit alerts to consumers or communicate with intelligent transportation systems, and the number of fatal accidents may decrease.

As a distinctly non-visual, low-cost, and mobile branch of vehicle recognition research, the smart sensor vehicle recognition system has the potential of contributing to the integration of intelligent transportation systems with human activity recognition, such that a person’s travel activities may be tracked in and out of their vehicle, and subsequently has the potential to contribute to and integrate with all other mobile-based activity systems. Expanding on the findings from Pias et al. [4], this paper contributes to vehicle recognition research based in smart sensors.

This study asks the following research question: can high accuracy be attained using only an accelerometer and gyroscope within smart phones to recognize key differences in the vibrations and movements of modes of transportation, in order to enable their real-time recognition? Specifically, this study aims to identify and distinguish these features among four specific modes of transportation: bicycle, automobile, train, and bus. Moreover, this model intends to maintain exceptional accuracy regardless of traffic and weather, as well as the make and model of the automobile or cell phone.

The paper is organized as follows:

1. A literature review details each branch of the vehicle recognition literature, with a focus on both theoretical and practical elements of these works. Each article of the literature review is examined for how the smart-device sensor model may benefit its practical utility.
2. Data collection, data processing, and our deep learning model and experiment are detailed.
3. Results are analyzed and explained.
4. A discussion with an emphasis on implications and future research is presented, noting a warning about the danger of driving while holding handheld smart devices.

## 2. Literature Review

**Figure 1:**
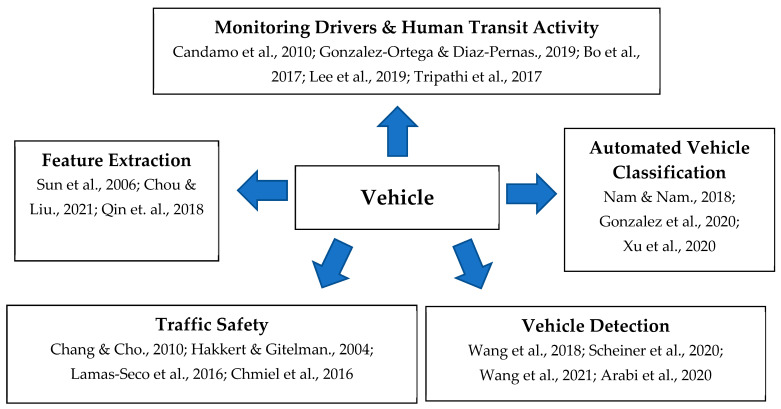
Vehicle recognition research outcomes.

Within the body of literature for vehicle recognition research, there are numerous pursued outcomes, as categorized by Figure 1. With vehicle recognition via smart sensors, it is possible to address these five functions of vehicle recognition research. When pursuing the functional outcomes of Figure 1, using smart sensors may provide results that could assist a wide array of transit and vehicle concerns, enhancing the capacity of intelligent transportation systems.

This paper proposes a system of vehicle recognition via smart device sensors. The smart device, when placed flatly on a seat inside of a moving vehicle, or when mounted, showed the ability to identify key vehicle features. When processed through our neural network machine learning system, this leads to accurate vehicle classification for cars, buses, trains, or bicycles under varying conditions.

**Figure 2:**
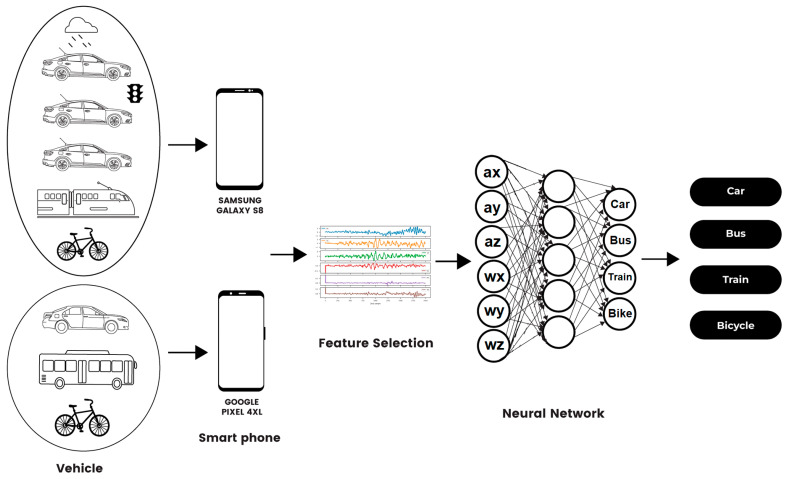
Our vehicle recognition pipeline.

In our model, shown in Figure 2, accelerometer and gyroscope data were taken from a bicycle, bus, and Toyota Camry into the Vieyra Software app on a Google Pixel phone for processing. Similarly, the same data from a bicycle, train, and Honda Insight were sent to the Vieyra Software app on a Samsung Galaxy phone for processing. In this second set, the Honda Insight was driven in the rain and in traffic, as well as on clear highway roads, for comparison. To determine that the vehicle set and smart phone model were not determining factors, the two sets were processed with the same feature selection and neural network, and ultimately categorized as car, bus, train, or bicycle. To test the placement of the smartphone, the device was located both on the seat of the Honda Insight, and additionally tested when securely mounted on a Vicseed universal dashboard cellular mount, with a two-step suction lock design. While the placement on the seat vs. the mount did not produce any differences in the outcomes, we discuss later the need for further research in how mounts affect smart device sensor vehicle recognition.

### 2.1. Vehicle Recognition via Smart Sensors

**Figure 3:**
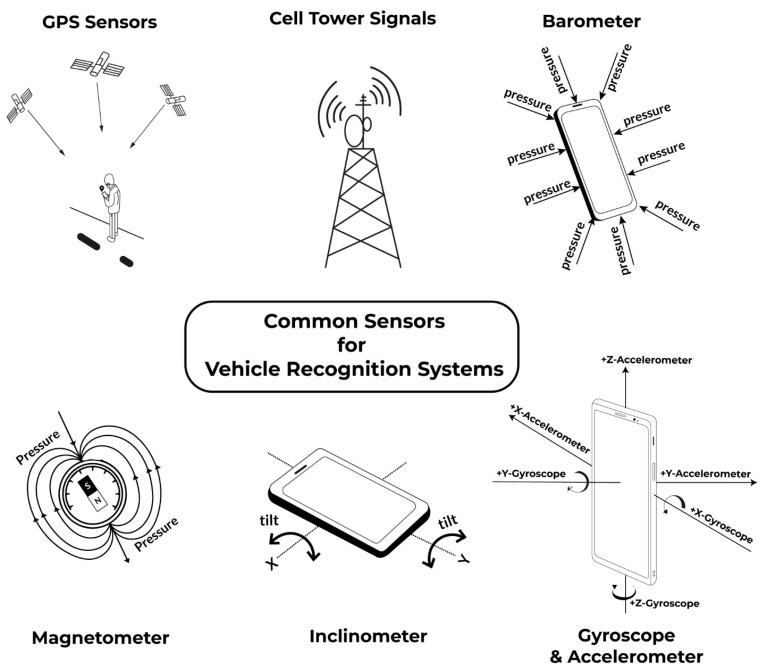
Common sensors for vehicle recognition.

Smart sensors, rather than just using cameras, take in numerous different kinds of sensing data, for which Figure 3 depicts some common examples. Often information from sensors are interpreted for further meaning by analyzing the raw sensor data through machine learning, deep learning, or neural network analysis. Numerous studies have used long-short term memory (LSTM) classification for smart sensor recognition for modes of transportation, such as in Ref. [5], which analyzed GPS, cell phones, and Wi-Fi receptors for determining whether subjects were still, walking, running, biking, driving a car, taking a bus, train, or subway. These authors observed between 88–94% accuracy on classifying each vehicular mode of transportation, with GPS proving to be their most valuable input. While Ref. [5] obtained an accuracy of 88–94% with the use of an LSTM classifier and three sensors, our study required only two sensors—the accelerometer and gyroscope—and attained a higher accuracy of 98%.

With deep convolutional bidirectional-LSTM, which is a method combining convolutional and bidirectional LSTM layers, Ref. [6] predicted whether users were traveling by car, bus, train, subway, walking, running, biking, or remaining stationary by analyzing the raw data of smartphone sensors. Accelerometer data, gyroscope data, magnetometer data, linear acceleration, gravity data, orientation (quaternions), and ambient pressure were utilized to achieve an F1 score of 0.96, but found that train and subway data were more difficult to distinguish, a problem that they were able to mitigate through the inclusion of GPS location data. Ref. [7] utilized LSTM to analyze Wi-Fi, GPS, LTE, and CDMA receivers from smartphones for identifying whether people were either not moving, walking, running, riding a bike, driving in a car, on a bus, on a train, or taking a subway. Four-fold shuffling was used to test the pipeline on the validation set with random shuffling, arriving at a best shuffle F1 score of 84.07%. Both Refs. [6,7] required a greater number of sensors, and also obtained lower accuracy than our 98% accuracy with only two cost-effective smart device sensors.

Ref. [8] used smartphone sensor data, including from accelerometer, gyroscope, barometer, and magnetometer sensors, to recognize whether users were still, walking, running, biking, or in a car, on a train, or on the subway. Combining residual and LSTM recurrent networks, their MSRLSTM model boasted accuracies of over 98% and recognition precisions greater than 95%. While this study boasts the same 98% accuracy that we attained, Ref. [8] added additional sensors, namely barometer and magnetometer sensors, which were not needed in our study to obtain the same level of accuracy.

A long short-term memory (LSTM) classifier analyzed raw data from smartphone accelerators, gyroscopes, magnetometers, and barometers to recognize transportation modes. Ref. [9] achieved 96.9% classification with only low-power-consumption sensors among 58 volunteers with android smartphones. While Ref. [9] attained a high accuracy of 96.9%, this was both slightly lower than our 98% accuracy and, more importantly, required four, as opposed to our two, sensors to arrive at these results.

Among numerous other studies that have used random forest (RF) classification for recognizing vehicles and travel modes, Ref. [10] used GPS, speed, and accelerometer sensors from six students to classify modes of travel, resulting in walking and biking accuracies of more than 90% with a univariate movelet approach, which segmented the accelerometer time series data into movelets and clustered segments of the same mode. RF sorting of the three modes of motorized transportation were classified, and proved to be the best with a 92% accuracy.

The smartphone GPS and accelerometer data of 50 participants were analyzed in such a way that incorporated a multinomial logit model into a RF framework. Ref. [11] significantly improved not only overall prediction accuracy, but also classification of less-represented modes of transportation. In another study using RF classification, 722 samples were collected for identifying vehicle classes of cars, buses, trams, and trains, from which GPS and accelerometer smartphone data were analyzed. Ref. [12] achieved a mobility detection accuracy of 87 % when using 10-fold cross validation on both a RF and an IBk algorithm. While all three studies [10,11,12] used a GPS sensor with a RF classifier, the higher accuracy in Ref. [10] can be explained by adding both speed and accelerometer sensors. However, even with their obtained 92%, it still realized a lower accuracy compared to our 98% using only accelerometer and gyroscope data. 

To determine the transportation mode of users, including standing still, walking, running, bicycle, motorcycle, car, bus, metro, train, or high speed rail, researchers collected gyroscope, accelerometer, and magnetometer sensor data in Ref. [13]. RF analysis yielded 83.8% accuracy, while applying a healing algorithm improved the accuracy to 93%. Here, introducing magnetometer data increased accuracy to 93%, but this was still lower than our model’s 98% accuracy. 

Using Google’s AutoML Tables service to preprocess and evaluate the Sussex–Huawei location transportation (SHL) dataset, Ref. [14] created 975 features by hand in order to achieve reasonable prediction quality. Smartphone GPS location data, GPS reception, Wi-Fi reception, and GSM cell tower scans were analyzed to determine whether the mode of transportation was standing still, walking, running, biking, driving a car, riding a bus, taking a train, or a subway. Incorporating the K-nearest neighbor classifier (KNN), RF, the extra trees classifier (ET), and the XGBoost classifier (XGB), Ref. [15] also took data from various radio sensors to interpret people standing still, walking, running, biking, driving a car, taking a bus, on a train, or subway. Their RF model presented their best results, with 93.4 % accuracy.

With Wi-Fi, Bluetooth, and GPS sensor data, Ref. [16] used RF, multinomial logistic regression (LR), and a support vector machine (SVM) to analyze daily transportation modes of four subjects, categorized as either self-powered (i.e., walk or bike), taking a bus, driving, taking a train, or riding the subway. With Wi-Fi and Bluetooth demonstrating a significant impact on model performance, and notably excluding accelerometer data, their model achieved an overall classification accuracy of 89% and a precision of 87%. In Refs. [14,15,16], the best accuracy obtained was 93.4%, which was lower than our results, while also using more sensors than ours to detect mode of transportation.

A transportation classification system demonstrated the capacity of GPS and accelerometer details from smart gadgets to identify the modes of user outdoor transportation. The system proved an accuracy rate of 96.31% when used on a dataset collected from 18 smartphone clients [3]. This study used the same number of mobile device sensors as our study, approaching but not quite reaching our 98% accuracy. In another study, road vehicle wheelbase and axle counts were acquired with piezo and magnetic sensors, leading to a near-instantaneous vehicle classification success rate of over 90% [17], an accuracy that was significantly lower than ours, while also using sensors that are also not as commonly available within handheld mobile devices.

Using rotation vector sensors, accelerometers, uncalibrated gyroscopes, linear acceleration, orientation, speed, game rotation vector, sound, and gyroscopes, Ref. [18] recorded activity data when subjects were standing still, walking, driving in a car, riding a bus, and taking a train. With an ensemble method of machine learning algorithms, KNNs, and random subspace, all bundled into stacked learning, they ultimately used a neural network architecture to successfully determine the transportation mode with an impressive 90% accuracy, though still decidedly lower than our 98% obtained with only an accelerometer and a gyroscope.

IoT sensors were shown to be effective for vehicle detection in Ref. [19]. Using infrared sensors, radar sensors, wireless magnetometers, and video image processors, researchers detected on-road vehicles with a 92.36% accuracy and 0% false alarms.

To distinguish between 38 users’ modes of travel, including being stationary, walking, motorized private transport (car or motorcycle), or public transportation (tram, bus, or train), Ref. [20] obtained over 30,000 observations from 30 different brands of mobile phones. Using GPS sensor, accelerometers, and GIS user contextual data, the authors achieved a mean accuracy of 96%.

Using accelerometer, magnetometer, and gyroscope smartphone sensors, Ref. [21] analyzed eight transportation modes, achieving 93.8% accuracy with an ensemble of machine and deep learning methods, as well as 97.2% accuracy when applying a Hidden Markov model (HMM). Evaluating accelerometer, gyroscope, and GPS sensor data with an ensemble method incorporating naive Bayes, Bayesian networks, kNN, logistic regression, J48, decision tables, and random tree models, Ref. [22] effectively detected walking, running, cycling, and traveling by bus or car with an exceptional accuracy of 99.5% and a precision of 99.6%, surpassing our own 98%, though still using a somewhat less cost effective three smart device sensors, as opposed to our two. Nonetheless, this is a very impressive model, demonstrating similarities to our own, which is limited to only sensors available in smart devices.

To distinguish between transportation on a bus, train, car, or subway, as well as walking and running, Ref. [23] analyzed smartphone data from accelerometer, gyroscope, magnetometer, and gravity sensors in their real-time trajectory segmentation method. Incorporating TSMs (transition state matrices) to detect the transport mode change point, they determined both vehicle identification and mode change points, with an overall accuracy of 98.52%, slightly higher than our own study’s results. Nevertheless, this marginal increase in accuracy required four sensors, rather than our more cost-effective two.

Ref. [24] successfully categorized a compact car, a truck, and a Hum V, as vehicles of varying weights, via a Fisher linear discriminate vector (FLDV)-based algorithm. While this study initially used seismic sensors (at less than 100 Hz) to detect acoustic sampling, their 99% accuracy was reported for an integrated license plate recognition system that used image processing from video cameras, rather than solely using sensors for their detection.

Accelerometer, gyroscope, and magnetometer sensor data from the android smartphones of 8 participants between the ages of 20 and 45 were collected over 79 h for training a vehicular activity detector and classifier [25]. Their multi-tier architecture, which included a segment-based approach called “healing,” detected travel mode among stationary, walking, car, bus, tram, train, metro, and ferry at 95% effectiveness. Our model achieved slightly higher accuracy for mode of transportation classification, and used a more cost-effective two, rather than three sensors.

With only smartphone accelerometers, Ref. [26] successfully generated a dataset for recognizing modes of travel for walking, cycling, taking a train, a bus, and taxi. This was achieved through the vibrations within the raw data, although their dataset study did not use machine learning to achieve a comparable experimental outcome.

Ref. [27] used a micro-electro-mechanical system (MEMS) of magnetic sensors with SVM to distinguish between heavy-tracked vehicles, light-tracked vehicles, and light-wheeled vehicles. With each vehicle passing by the sensor at a uniform speed, they achieved recognition rates of more than 92% for heavy-tracked vehicles and light-tracked vehicles, and 89.5% for light-wheeled vehicles. This study, using sensors located outside of the vehicle and not from common mobile devices as in ours, still obtained a lower accuracy than our 98%.

In 2021, Li et al. collected only GPS and Wi-Fi data from people standing still, walking, running, biking, driving a car, riding a bus, or taking a train or subway [28]. Using convolutional and recurrent neural networks, features were extracted, and long-term temporal information was captured. Results showed that GPS consistently outperformed Wi-Fi, and subway activity was best recognized via location data. However, accuracy ranged only between a relatively low 34–48%.

By fusing geomagnetic sensors and frequency modulation radio data, Ref. [29] scored a vehicle detection accuracy of 95.4% on high-chassis vehicles, or buses, passing by the sensors at high and low speeds of 10 km/h and 60 km/h.

Accelerometer, gyroscope, and magnetometer sensors were used for interpreting driving activities, including stopping (S), going straight (G), turning left (L), or turning right (R), while either walking, biking, motorbiking, driving a car, or riding a bus [30]. Authors achieved an accuracy of 98.33% in detecting vehicle modes, and an average accuracy of 98.95% for identifying driving events. In another study using accelerator, gyroscope, magnetometer, and barometer sensors, Ref. [31] distinguished between bus, car, metro, and train transportation modes. The authors’ CNN algorithm had the highest accuracy of transportation mode recognition when based on a Keras framework, yielding an accuracy of 94.2%. While the results of Refs. [29,31] had lower accuracy than ours, Ref. [30] was more comparable to the success of our model. However, this slightly higher accuracy required additional magnetometer data [30] to our more cost-effective accelerometer and gyroscope model.

To identify between users remaining stationary, walking, running, cycling, driving a car, riding a bus, taking a train, or riding the subway, Ref. [32] analyzed GPS, Wi-Fi, and cellular data with three different tree-based models. Their model had a relatively high accuracy for bicycle data with an F1 score of 0.82, but alternatively, a low F1 score of 0.25 for running. In another study using an ADXL345 accelerometer sensor to read vibrations on railroad tracks, Ref. [33] were able to detect the arrival of trains at an average accuracy of 83%, a significant improvement over similar previous studies. Impressively, Ref. [33] obtained its results with only accelerometer data, but it did so only by identifying train movement along railroads, and at a markedly lower accuracy than our 98%.

Fusing data from the accelerometers and gyroscopes of mobile phones and smart watches, Ref. [34] utilized a better-than-the-best fusion (BB-Fus) algorithm to distinguish between meeting-walking activity combinations and meeting-motorized transportation activities. With an SVM classifier, their unique model’s overall classification performance hit 98.32%. Raw data from mobile accelerometer and gyroscope sensors for 20 users with sampling rate of 50 Hz were analyzed by CNN [35]. When categorizing bus, car, subway, and train travel modes, they achieved an accuracy of 91%. Refs. [34,35] notably used the same exact combination of sensors as used in our study. While Ref. [35] obtained a noticeably lower accuracy, Ref. [34] obtained an almost equivalent result to ours. Our study, however, ensured that obtained accuracy was consistent across different traffic and weather conditions, although their introduction of a BB-Fus algorithm did provide slightly better performance than our model by 0.32%.

Ref. [36] examined GPS location, GPS reception, Wi-Fi reception, and GSM cell tower scans from smartphone sensors to perform transition-points-based segmentation. They divided types of transportation into two groups based on speed, and subsequently applied a high performance optimized XGBoost classifier, ultimately obtaining an 88% accuracy.

GPS, Wi-Fi, accelerometer, and cell-ID information were used to identify departure and arrival times, origins, destinations, modes, and travel purposes of around 800 respondents. Ref. [37] correctly classified over 80% of trips, and this increased up to 95% for distances greater than 20 km. One problem that comes with the use of cell-ID information in [37] is that it leads to privacy intrusion, as the data are acquired from, for example, call records of the user.

A combination of smartphone sensors, including GPS reception, GPS location, Wi-Fi, and GSM tower scans data were analyzed with a combination of convolutional subnets to extract local features, as well as a transformer subnet that captures long-term dependencies. Ref. [38] distinguished between users standing still, walking, running, cycling, driving a car, or riding a bus, subway, or train to yield a best F1-score of 0.8779. The use of GPS sensors in [36,37,38] provides less specific displacement sensitivity than the gyroscope and accelerometer used in our study, a difference that can at least partly explain our higher accuracy of 98%.

Inertial and pressure sensors, accelerometer, gyroscope, magnetometer, linear acceleration, gravity, orientation, and ambient pressure data were recorded from smartphones by Ref. [39] when determining which of the eight transportation modes (still, walk, run, bike, car, bus, train, and subway) a user had used to travel. The performance of the respondents had a wide range, from 53.2% to 93.9%, although 16 out of 19 respondents had over 90% accuracy. This study’s best accuracy of nearly 94% was still lower than our 98%, and used seven more sensors than our model.

The novel Spiderwalk vibration sensor worn inside the subject’s shoes, under the feet, was used to collect data over one month from six subjects. Transmitted wirelessly through Bluetooth connections to their smartphones, Ref. [40] demonstrated a high detection accuracy of 93.8% for determining the kind of vehicle a subject was traveling in, or if the participant were walking or sitting, and on what kind of surface. While the Spiderwalk method obtained a good accuracy of 93.8%, it required a specialized sensor in people’s shoes that is not readily and passively available via people’s existing smartphones, as our model does.

Ref. [41] classified walking, cycling, e-bicycle riding, riding a bus, and driving a car via smartphone GPS, accelerometer, GSM, and Wi-Fi sensors. Overall, their accuracy exceeded 93% for both the training and test datasets, with precision and recall exceeding 80% for each mode of travel. GPS location, GPS reception, Wi-Fi reception, and GSM cell tower scans were analyzed via a LightGBM (LGB) classifier to classify eight different transportation modes. With an F1 Score of 0.665, Ref. [42] found that smartphones on trains, buses, and subways were more distinct with their model than in other modes of transportation that rely on user movement. For both [41,42], four sensors were used to obtain markedly lower accuracy than ours, which required only two sensors to recognize and classify modes of transportation at 98% accuracy.

To recognize cars, minibuses, buses, and trucks, as well as the direction of a passing vehicle, Ref. [43] worked with wireless magnetic sensors to detect and distinguish between the metallic parts of those vehicles. When identifying vehicles passing, their paper had a general accuracy rate of 94%. The classification of vehicles had accuracy of up to 100% in trucks, although they experienced a reduced accuracy of 92% when distinguishing between minibuses and buses. While this study did obtain 100% accuracy in trucks, our model’s 98% accuracy was applicable to more modes of transportation. Furthermore, the wireless magnetic sensors were not handheld smart device sensors, capable of also passively integrating with other aspects of human activity detection, as can be obtained through accelerometers and gyroscopes.

Classifying cars, vans, buses, and trucks on a two-lane road, Ref. [44] used magnetometer sensors, identifying a RM3100 as the best performing sensor, due to its high sensitivity and low noise. Ref. [45] tested axle-detecting sensors for vehicle recognition. They found that mounted free-of-axle detector (FAD) sensors often failed to identify vehicle axles, if there was either bridge vibration, multiple vehicle presence, or a deviation of vehicles transversely in their lane. They instead proposed a wavelet-based approach, with Shannon entropy and a correlation factor, to improve axle detection.

Prior research has also found that significant losses in cost effectiveness are incurred by smartphones using a greater number of sensors, and that an effective way of saving energy in smartphones is to turn off those sensors that are not being used [46,47,48]. For example, Ref. [47] found that an idle smartphone lasted 51.27 h, while a smartphone with an accelerometer lasted 31.51 h, a significant reduction. A smartphone with a gyroscope running lasted just 28.15 h; and a smartphone with a magnetometer running lasted 34.45 h. The GPS was the most energy consumptive, lasting only 17.42 h.

The energy consumption of various smartphone sensors was assessed for percentage of consumption by Ref. [49], and they found that the accelerometer, when applied in outdoor motion, used roughly two-thirds as much energy consumption at 8% as the magnetometer with 12.5%. The GPS sensor was found to use a sizable 53%, and the inclinometer used 5.9%. For network interfaces, the same study [49] found that, when moving outdoors, Wi-Fi sensors used 32% of energy consumption, while cell tower sensors used 20%, and Bluetooth used 28%.

Another study [50] looked at the use of Wi-Fi and GPS, along with other major functions, and found that battery times when each sensor was active were relatively consistent across makes and models of smartphone. Stronger Wi-Fi signals also did consume more energy than weaker Wi-Fi signals. This study [50] also found that GPS sensors used a very sizable portion of battery life, being the costliest use, in terms of battery consumption. This study also looked at accelerometers, magnetometers, and gyroscopes, concluding that the magnetometer was the least energy efficient sensor among those three.

To summarize, a review of recent literature on mode of transportation recognition performed via smart sensors yields only a small number of articles that attempted a method which was as cost-effective as ours. Most notably [26,33,34,35], only Ref. [34] achieved the near-perfect accuracy that our results provided, and that article did not test for consistency across traffic or weather conditions as ours did. As listed in Table 1, other studies obtained incredible accuracy, but were either not as cost effective by using more sensors [21], or were not integrated with common handheld devices [9], had significantly lower accuracy [35], or were not as generalizable in its accuracy to multiple modes of transportation [43] as ours.

### 2.2. Intelligent Transportation Systems (ITS)

Vehicle recognition via smart sensors has the potential to integrate the newer technologies and advancements for intelligent transportation systems. Safety in vehicles has dated back to when seat belts and windshield wipers were first introduced. Crossing guards and stop signs helped signal to people whether to go or stop leading up to the traffic lights, and more recently, red light camera technology has been introduced. Previously a far-fetched objective, red light cameras are now a normal installation at major intersections, resulting in an 18% reduction in car and motor vehicle collisions, leading to less traffic injuries and fatalities [51].

**Figure 4:**
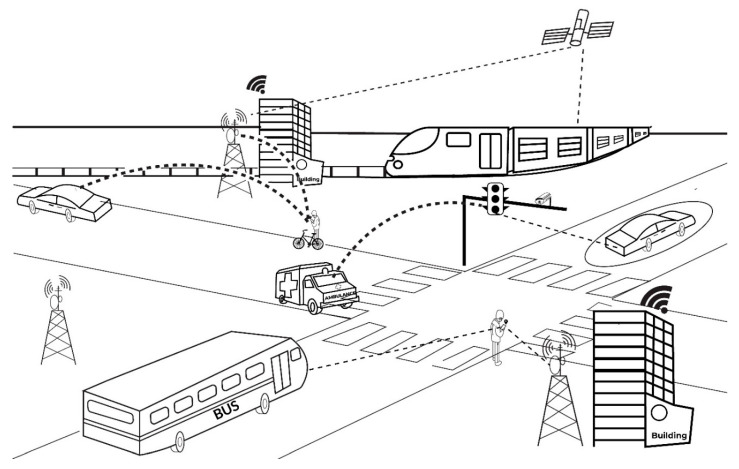
Intelligent Transportation systems with smart sensor transportation recognition.

As shown in Figure 4, vehicle recognition via smart device sensors can help enhance the already growing integrated body of artificial intelligence and vehicle recognition technologies that stand to advance traffic management and public safety, in addition to vehicle and surveillance technology. Additional safety features, such as tracking car movement to identify potholes, when suspension systems malfunction, or when there is excessive speeding, can be directly communicated in the future if wireless technology that verifies motor vehicle operation from the interior of the vehicle can make a direct communication with such features. Moreover, the ubiquity of smart phone use in society [52] suggests the potential to overcome the proximity limitations of intelligent transportation systems dependent on systems such as red light cameras, tying the surveillance of traffic incidents literally to the availability of a camera-equipped traffic light at the time of the incident [51].

Figure 4 details the integration of not-so-futuristic technologies suggested by this paper’s review and experimental study. The GPS satellite in Figure 4 connects with and recognizes the train along the railway, potentially helping to identify its arrival time and speed. This can alert the traffic system to prepare the traffic lights and road signals, informing the bicyclist and car to the potential approach.

The ability of smart device sensors to connect and integrate with other sensors within an intelligent transportation system network, therefore, creates the potential for higher levels of interconnectivity, information, and interactivity throughout the transportation grid. Driver behavior can be tracked by smart phone sensors, with the potential of understanding the cause of, or even avoiding accidents [53]. A pedestrian is also able to know in real-time the arrival time of the bus and train. The traffic light can anticipate the pedestrian’s and bicyclist’s approach, potentially readying its signal to best accommodate them. A smart road can identify, track, and interact with the pressure or magnetism from the connected car above it, monitoring and interacting with vehicles in real-time. In the communication between all these modes of transportation, the traffic light and public transportation system, as well as any potential autonomous vehicles, are able to adjust to traffic conditions and/or accommodate the emergency vehicle’s need to race ahead and bypass any traffic conditions. Smart buildings, as shown by the corporate offices in the system, are similarly connected to all these systems, allowing them to adjust to changing conditions if any advantages can be elicited from doing so. Lastly, the entire grid of the urban center is connected to all other urban centers, surrounding traffic lights, bus stops, and train terminals, by the ubiquitous connection of stop lights, cell phone towers, people carrying devices with smart sensors, and GPS satellites, to better control and automate intelligent transportation systems.

Accidents, violence, suspicious activity, terrorism, and vandalism are all events for which transit systems are constantly seeking better ways to prevent and respond. Candamo discussed existing state-of-the-art techniques for automated activity detection mechanisms, with an emphasis on human activity monitoring in transit applications. A literature review of 52 publications analyzed strengths and shortcomings, with the main goal of the review being to supply field researchers with information of progress made thus far, and to help in the identification of areas in which more research is needed [54]. Such surveillance would benefit from casting a larger net to spot negative behavior [55,56], something that further research on smart device sensors with intelligent transit systems may address.

On the first page of the 2010 Official Journal of the European Union [57], “Intelligent Transport Systems (ITS) are up to date applications which… allow different users to be better educated so that they can use transportation networks in a safer, more organized, and “smarter” manner”. Intelligent transportation systems (ITS) is an inclusive term that involves a wide variety of systems and software applications aimed at enhancing road safety and performance. The authors of one study assessed vehicle category by concentrating on obtaining correct speed and wheelbase measurements. Vehicle classification was dependent on the vehicle’s wheelbase dimensions, as well as the number of axles and metal weights. A complementary magnetic sensor, as well as a camera to check the experiment’s results, are included in the unit, which aids in categorizing the car when the wheelbase measurement is representative of many categories. Tested in real-world traffic, the system is simple to set up and non-invasive to use, and it has a classification success rate of over 90% [58].

Multimedia systems, such as *INSIGMA* [59], which utilize various sensors to simultaneously monitor and control urban transportation can potentially benefit from the integration or interaction of smart device sensors. Similar to red-light cameras, these systems can be limited to use in traffic centers, such as urban settings, due to the availability of surveillance systems and convenient places they may be installed. With the availability of smart device sensors in automobiles, smart watches, and smart phones, it may be possible to incorporate multi-sensor monitoring systems into even rural areas, where cell phone reception is available, though complex urban ITS systems are not. Another study obtained concurrent vehicles’ inductive signatures (SiDIVS) while driving [60]. Their results showed a strong correlation between the signatures acquired from similar cars and an important distinction between the signatures relating to different vehicles. This allows for better tracking of vehicle occupancy, speed, type, and density, systems that also may be corroborated by a driver’s personal smart device sensors.

In another study [54], researchers monitored Shimmer physiological sensors while synchronizing its operation with a driving simulator. The Android app tracked drivers and evaluated the relationship between their physiological states and driving efficiency. Electromyogram (EMG), electrocardiogram (ECG), and galvanic skin response (GSR) accelerometers and modules, as well as a gyroscope and magnetometer, were configured, selected, received, processed, graphically represented, and stored in the app. Among 25 people, significant associations were discovered between the EMG, ECG, GSR, and gyroscope sensors, including that of sensor data with traffic offenses and car details.

Another study looked at methods for detecting and classifying vehicles using thermal cameras and visible light. When validated experimentally, the methodology achieved an accuracy of 92.7% for the visible classifier, and 65.8% for the thermal classifier when the cars were categorized into various types for example SUV type, RV type, and sedan type [61].

Non-line-of-sight (NLOS) techniques are used to retrieve information about obstructed items from indirect shadows or reflections that are also in the detector’s direct view [62]. Previous NLOS techniques have difficulty beyond regulated laboratory settings, as well as with large-scale outdoor areas and extremely fast movement, as seen in traditional automotive scenes. The use of automotive Doppler radar sensors to identify and monitor features in large-scale complex scenarios out of the straight line of sight was demonstrated in this paper. The proposed solution was shown to work in real-world autonomous driving scenes, allowing for bumping alerts for pedestrians and cyclists before they were detected by current direct line-of-sight sensors.

With a model based on the computationally efficient algorithm to reconstruct vehicular traces (CERT), a novel algorithm that matches data from accelerometers and magnetometers to real roads, Ref. [63] identified users’ paths traveled. They achieved nearly perfect path recognition for medium and small-sized cities, and, in particular, the longer the user’s path, the easier it was to accurately identify it.

Evaluating D3, a fine-grained system which informs drivers of their abnormal driving behaviors which may otherwise be ignored, Ref. [64] aimed to improve driver awareness and safety. Incorporating accelerometer and orientation sensors from 20 drivers with distinct vehicles in real driving environments, researchers identified specific abnormal driving behaviors in real time, with an average total accuracy of 95.36%. If these systems were integrated with customized apps that work with smart device sensors, this may enable real-time notifications, emergency calling, and enhance the ability to recognize and respond to pre-crash conditions.

Ref. [65] designed an internet of vehicles system to automatically reduce road congestion by diverting cars toward the path with the least traffic. The system used GPS, internet of vehicles sensors (IoV), stationary sensors, and visual sensors to recognize vehicles, map the best possible trajectories to avoid traffic, and then control lights at intersections, achieving a vehicle classification accuracy of 95.02% and a traffic reduction rate of 10.31%.

A total of 20 participants aged from 25 to 55 and all with valid driver’s licenses, wore a wrist-mounted inertial measurement unit, consisting of accelerometers, gyroscopes, and magnetometers during driving trials involving intermittent planned distractions. Ref. [66] found that their non-camera, real-time driving behavior monitoring and mitigation was an effective prototype for averting hazardous driving behaviors in real time without camera-related privacy concerns.

Focusing on preventing car accidents while overtaking on a two-lane road, Ref. [67] analyzed data from LiDAR sensors, radar, and cameras. Their model proved capable of assuring drivers were overtaking safely, even at high speeds.

An AI smart vehicle system proposed by Ref. [68] used wireless sensors to effectively achieve driver recognition and authentication, assist driver state monitoring, passenger counting, and unattended child detection, checking wirelessly obtained vital signs against their multiple-driver radio biometric database.

In their video measurement system (VMS), Ref. [69] used magnetic sensors, infrared sensors, photoelectric sensors, Doppler and radar sensors, inductive loops, and video camera systems to analyze traffic flow and speed measurement. While the system integrated visual detection as part of the vehicle identification mechanism, it used multiple low-cost sensors to integrate into existing intelligent transportation systems to control traffic.

Focusing on smartphone accelerometer data, Ref. [70] successfully classified six human activities, including standing, sitting, laying, walking, walking upstairs, and walking downstairs among 30 people aged 19 to 48. They found that the stationary activities of standing, sitting, and laying had a 0% classification error, and felt that inclusion of gyroscopes may have benefited their study further.

The combination of human activity detection [71] with vehicle recognition data may lead to a better understanding of driver behavior, as well as its effects on vehicle movement and traffic incidents. When intelligent transportation systems and vehicle detection mechanisms operate entirely from cameras and/or satellites operating outside of the vehicle, it may be difficult to precisely analyze the operational and behavioral decisions of humans inside the car. Was the driver having an argument with the passenger? Was the driver distracted by a phone call or a spilt coffee? The many sensors inside the phone may eventually be able to combine to forge a deeper understanding of the intricacies of these kinds of situations, which may lead to better understanding of accidents, traffic incidents, and their avoidance. Furthermore, the utilization of multiple sources of vehicle recognition data, as well as the standalone benefits of onboarding [72,73], suggests the potential for increased accuracy in both intelligent transportation systems, when incorporating smart device sensors into these systems.

Many advantages for vehicle recognition, traffic monitoring, and surveillance extend from an understanding of automated sensing. Few studies have considered the economics of vision detection deployment and performed a cost trade-off analysis [74]. With smart device sensor vehicle recognition is the ability to utilize the user’s existing technology, which may offset potential implementation and maintenance costs, since the end-user’s personal device would actually be sensing hardware utilized for vehicle recognition.

With intelligent transportation systems, and in each of the above studies, it can be understood that combinations of sensors, and especially incorporating sensors that can be carried on and interpret the behaviors of the driver, can add significantly to the functional utility of vehicle recognition systems. Smart device sensors, therefore, can become the connection between systems that perform vehicle recognition with intelligent transportation, smart vehicles, and tracking systems that intuitively understand the vehicle identification, path traveled, driver behavior, and behavior of the user before and after leaving the vehicle. This helps create a profile of the user that can ultimately lead to a better understanding of vehicle use, vehicle selection, and the users of vehicles. In the following sections, the methodology of smart-device sensor vehicle recognition research is detailed.

## 3. Methods

### 3.1. Explanation of Accelerometer and Gyroscope Technologies

**Figure 5:**
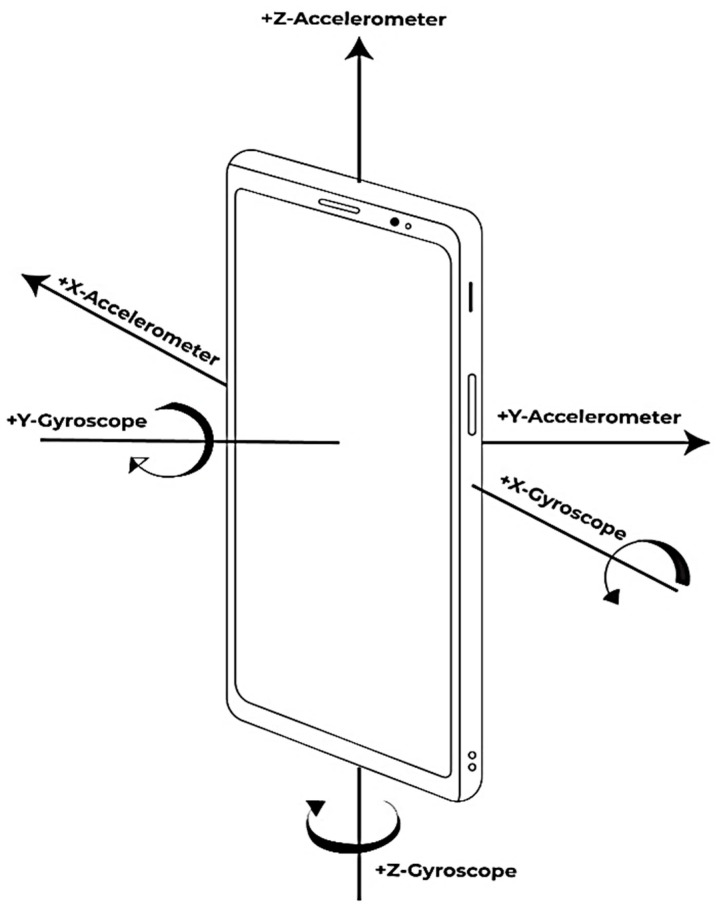
Gyroscope and accelerometer visual description.

An accelerometer records a subject’s entire acceleration profile generated as a result of movement or tilt due to gravity [75], as shown in Figure 5. Accelerometers can be divided into two types, based on the equipment used [76].

Piezoelectric accelerometers are further categorized into two groups based on different configurations. The first group includes Cantilever beam/piezoresistive/strain gauge accelerometers [75]. This group has a seismic mass as well as a piezoelectric component. The seismic mass is usually suspended close to the piezoelectric element. As movement occurs, the piezoelectric part deforms due to the acceleration heading towards the sensitive axis. This deformation appears in the form of bending, and results in a buildup of charge close to the piezoelectric element. The second group includes the compression-based piezoelectric accelerometer. The principle of this accelerometer is similar to that of strain gauge accelerometers, with the exception that the seismic mass is located above the piezoelectric part. In this case, movement in the related direction causes acceleration, which causes deformation, bending, and charge build-up in the piezoelectric element.

Capacitive accelerometers are commonly used in smart phones, cameras, and gaming systems due to their precision, user-friendliness, and absence of temperature calibration [75]. Seismic mass is caught in the middle of fixed anchor arms in this case. The floating arms of the seismic mass are at a specific distance from the fixed arms, while the seismic mass is in equilibrium (no motion/acceleration). When the sensor is accelerated, the suspended seismic mass shifts, as do the sensor’s floating arms. The distance between floating seismic mass arms and fixed arms (arms on fixed plate) changes as a result of this. The capacitance (ratio of electric charge/potential charge), which is used to calculate acceleration, changes as the distance changes.

Gyroscopic technology is also found within smart devices, and is the second major smart device sensor utilized in our research. The gyroscope senses an object’s vibrations and its Coriolis force, allowing it to interpret the angular velocity, thus creating the outputs that we use to understand and make various applications [77], as well as activity recognition [78,79].

### 3.2. Experimental Setup

In our model, accelerometer and gyroscope data were collected by the Vieyra Software app on a Google Pixel smartphone for the bicycle, bus, and Toyota Camry 2005. Accelerometer and gyroscope data were collected by the Vieyra Software app on a Samsung Galaxy phone for a bicycle, train, and Honda Insight 2013. In our choice of automobile, we determined no difference between the recognition of a non-plug-in hybrid vehicle (Honda Insight 2013) and a standard gasoline powered vehicle (Toyota Camry 2005). However, further research would be needed to determine if a completely electric plug-in vehicle was also equivalent.

Data were taken from the two cell phones onboard the vehicles. A total of two types of devices were used to diversify and mitigate the bias of specific mobile devices. In the Samsung Galaxy set, the Honda Insight was driven in the rain and in traffic, as well as on clear highway roads, for comparison. To determine that the vehicle set and smart phone model were not determining factors, the two sets were processed with the same feature selection and neural network, and ultimately categorized as car, bus, train, or bicycle.

A fixed time stamp was used to obtain the gyroscope and accelerometer values. The Physics Toolbox Sensor Suite by Vieyra Software, a software developed in part by a National Science Foundation grant for STEM Education, intended to display, record, and export data from smartphone inertial sensors was selected to obtain our smartphone data on both devices [80].

The routes and times of day were carefully selected to avoid any congestion, except in the one route that was specifically selected to test the model’s utility along urban stop-and-start traffic. In all other instances, we chose a straight and open road with a lower probability of a traffic jam. In another instance, heavy rain and flooding along the highway was also measured to ensure that severe weather was not a factor in altering the recognition of correct vehicle identification using our algorithm.

**Figure 6:**
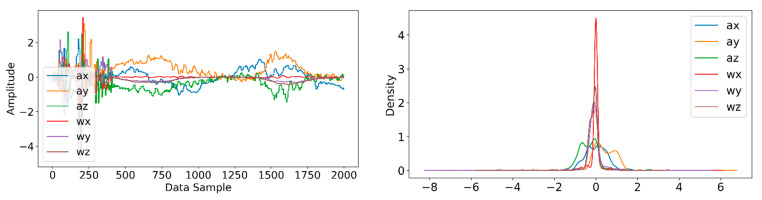
Raw sensor signal for Toyota Camry (**left**) and KDE plot (**right**).

**Figure 7:**
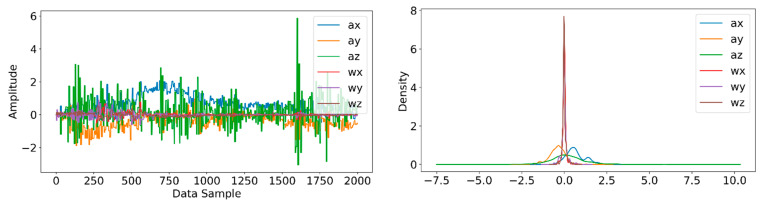
Raw sensor signal for the bus (**left**) and KDE plot (**right**).

**Figure 8:**
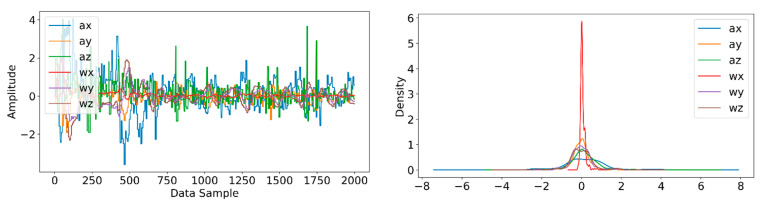
Raw sensor signal for the bicycle (**left**) and KDE plot (**right**).

**Figure 9:**
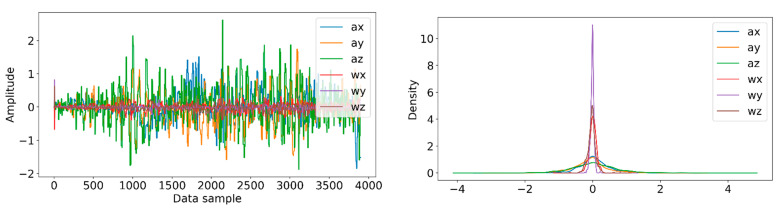
Raw sensor signal for the rail (**left**) and KDE plot (**right**).

Information from every vehicle type was obtained for 5 min each at separate times. A 3-axis gyroscope and accelerometer were used. The x, y, and z figures of the gyroscope, as well as that of the accelerometer, were collected. In our dataset, we therefore had six columns of information. Each time stamp represents a point in the data. Figure 6, Figure 7, Figure 8 and Figure 9 visualize the raw sensor signals for the Toyota Camry, Honda Insight, bus, and bicycle, respectively. The data are labeled with the same abbreviations as listed in the training data set, and color coded to highlight differences among the various vehicles more easily. Both car models were registered as “car”, and were easily distinguished against bus, bicycle, or train.

In Figure 6, when evaluating the Toyota Camry, no differences were found between the Camry and the other vehicle tested during our study, the Honda Insight. Both vehicles registered identically as “car”, and shared equal sensitivity in being distinguished against bus, bicycle, or train.

In Figure 7, the bus consistently showed as being distinctive, and was identified with high accuracy against car, bicycle, or train. Both car models were equally distinctive against the bus. In Figure 8, the bicycle consistently showed as being distinctive, and was identified with high accuracy against car, bus, and train. Both car models were equally distinctive against the bicycle.

In Figure 9, the train, for which we tested the Newark Light Rail line from NJIT Campus in downtown Newark, New Jersey, to its final stop on Bloomfield Avenue, in Bloomfield, New Jersey, consistently showed as being distinctive, and was identified with high accuracy against car, bus, and bicycle. Both car models were equally distinctive against the train (rail).

**Figure 10:**
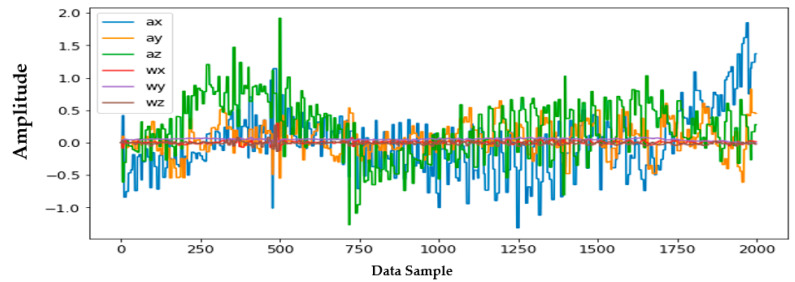
Honda Insight—clear highway.

**Figure 11:**
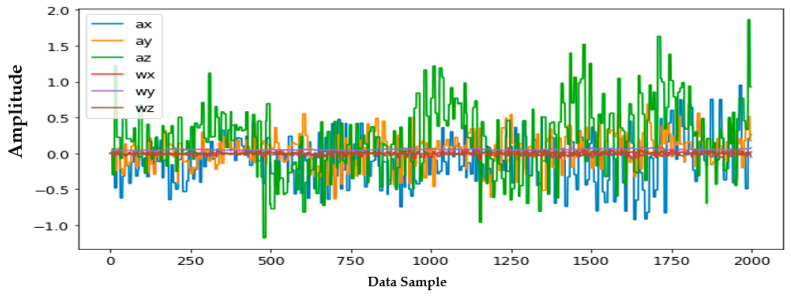
Honda Insight—in heavy rain.

**Figure 12:**
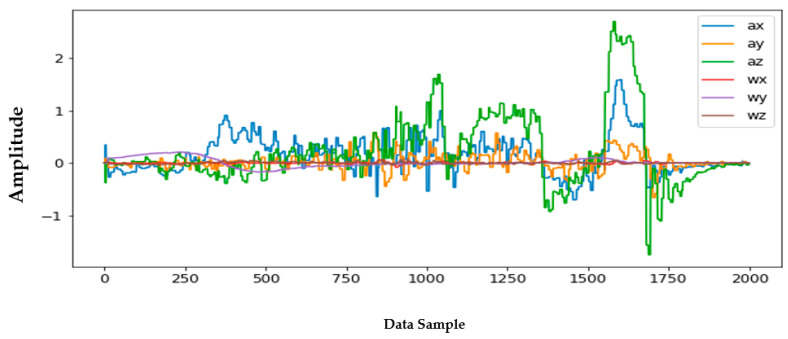
Honda Insight—in Newark heavy traffic.

In Figure 10, Figure 11 and Figure 12, no difference was found between the analyses using the car on a clear highway vs. the car in heavy rain vs. the car in heavy traffic in downtown Newark. Thus, neither weather nor traffic conditions affected the consistent identification of the vehicle using our algorithm during our study.

**Figure 13:**
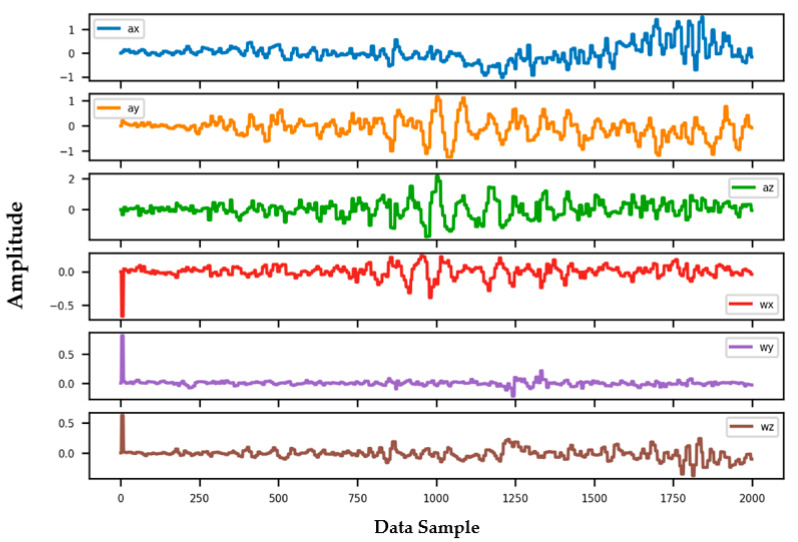
Raw data over time domain—separated plots for each vehicle.

Figure 13 shows the raw data for each of the vehicles over the time domain. The different colors represent three separate classes. The experimental setup, training model dataset, and raw data were all necessary to implement within the artificial neural network that constitutes the algorithm driving this study on vehicle recognition.

### 3.3. Data Preprocessing

**Figure 14:**
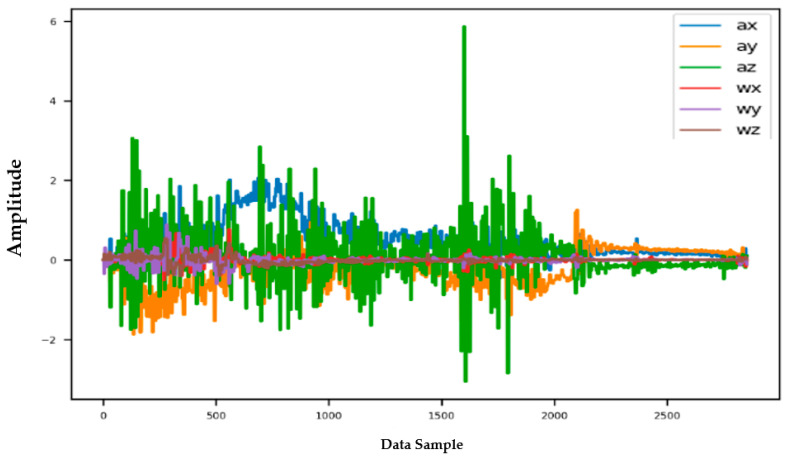
Data range: 0:3000 (input).

**Figure 15:**
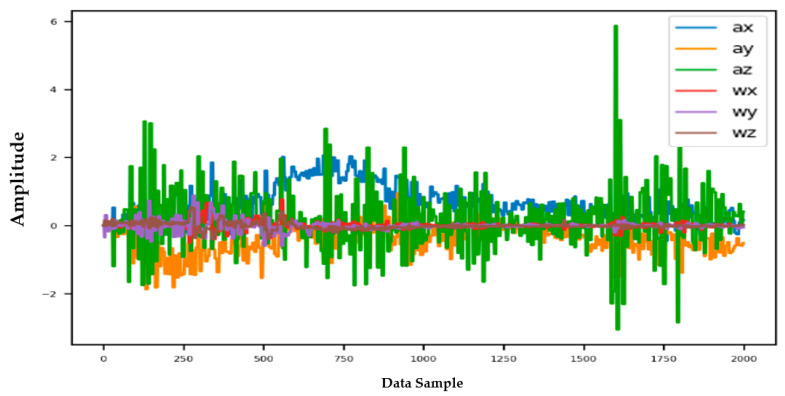
Data range: 0:2000 (output after step 1).

**Figure 16:**
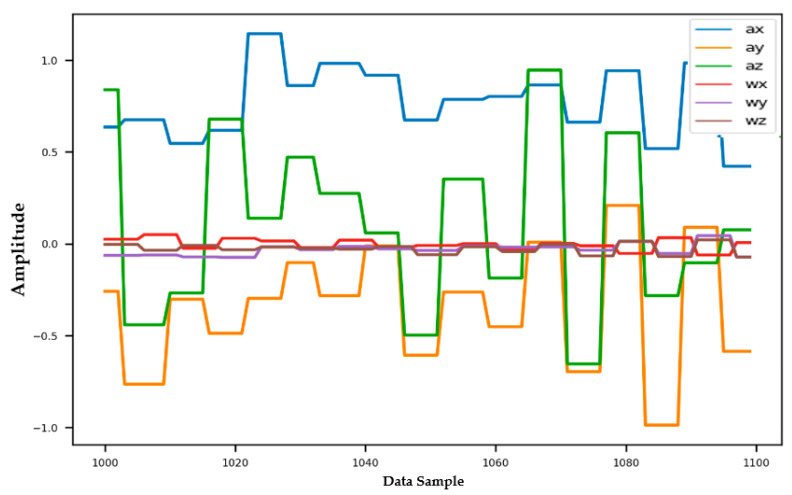
Data range: 1000:1100 (output after step 2).

Figure 14, Figure 15 and Figure 16 show the preprocessing of the data, as it is initially input in Figure 14, and then passes through the following steps 1 and 2 respectively:

1. The raw dataset for each class has different lengths. As such, all of them are trimmed to have the same length to reduce bias. The number of data points is considered to be 2800. Therefore, the dimension of each class data after trimming is (2800, 6), where 6 is the data channel.
2. Then, this trimmed dataset is segmented into snapshots using a sliding window of the length of 100 samples, and the stride is equal to 1 sample. After this step, the data dimension of each class is (2700, 100, 6) where 6 is the data channel, 100 is the data snapshot, and 2700 is the number of snapshots. In other words, the data of each class can be considered as a set of images, where each image dimension is (100, 6), and there is a total of 2700 images in each set.
  [More details about the sliding window: https://www.ibm.com/docs/en/mapms/1_cloud?topic=detectors-window-size-scheduling] (accessed on 1 April 2022).

### 3.4. Dataset Preparation

Each class data are concatenated to make a complete set of all classes. Thus, now the full dataset dimension is (10,800, 100, 6), where 10,800 = 2700 × 4.

Labels are created for each class, where car = 0, rail = 1, bus = 2, and bike = 3. In addition, one hot encoding is used, as they are similar to categorical classes. For example, the bus class will be represented as [0, 1, 0, 0].

Now, this dataset is divided into the train set (70%) and the test set (30%) randomly. The train set dimension is (7560, 100, 6), and the test set dimension is (3240, 100, 6).

## 4. Results

There can be different types of datasets; for example, tabular, image, and time series. Each type of dataset has a different orientation and dependence. Generally, the features of tabular data are not directly dependent on each other. However, the case is different for image and time series data. The image data are represented using tensors, and each row/column is dependent on another row/column. It means one column or row cannot be swapped with another column or row. The time series data are more complex than regular tabular data. They have data dependency. For this reason, general machine learning algorithms are not effective enough. 

As opposed to deep learning methods, machine learning algorithms require representative feature vectors as input. However, deep learning algorithms can use raw dataset for processing. It is very difficult to extract features from time series data. Statistical values can be extracted from time series data to train machine learning classifiers [81]. However, these feature sets are inadequate to represent the complete time series. As a result, the performance degrades. 

On the other hand, deep learning methods are free from these issues. In particular, LSTM and CNN have proved to be effective for dealing with such datasets [82,83]. LSTM works to identify data dependency where CNN uses convolution to extract features. The convolution operation of CNN is equivalent to Fourier transformation, which shifts the time series data to the frequency domain from the time domain. The frequency domain is more representative than the time domain. For this study, we utilized the CNN and its convolution property to learn the inherent pattern from a time series dataset. To evaluate our theory, we trained a number of traditional machine learning classifiers, as seen below in Table 2.

**Figure 17:**
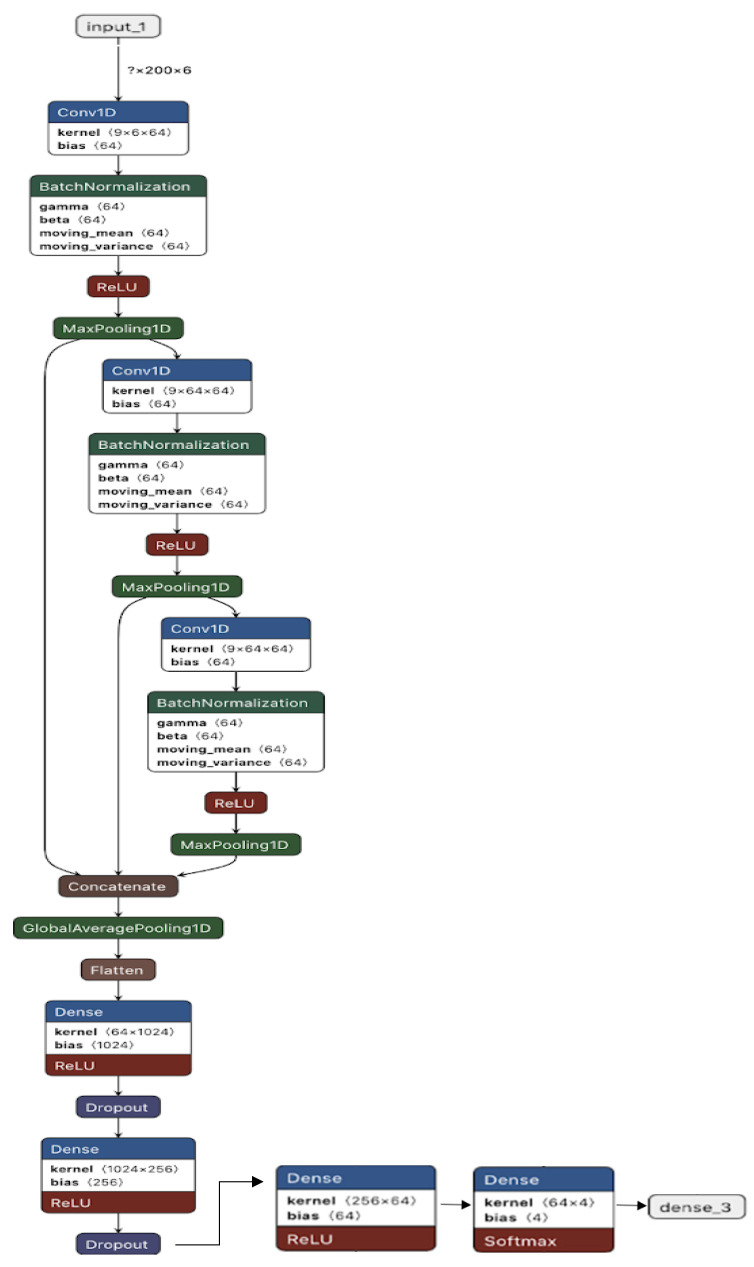
Our CNN model.

The machine learning models could not achieve a high accuracy compared to CNN. From this result, it can be safely assumed that the dataset is too complex for traditional machine learning models. Moreover, our proposed CNN is effective enough to classify the dataset with a very high accuracy. Therefore, we used 1D CNN for feature extraction. While LSTM models are good for time series data, new research shows that CNN models can sometimes be more effective in recognizing time-series patterns. In fact, 1D CNN has been proven to be very effective for pattern recognition from sensor signals. Ref. [84] uses 1D CNN to classify emotion patterns from EEG signals, and Ref. [85] also utilizes 1D CNN to detect arrhythmia from ECG signals with very good performance. All of this evidence from the literature provides a shred of strong evidence that 1D CNN coupled with MLP (ANN) can be very effective for pattern recognition from sensor signal data. The sensors generate signals which are captured in the time domain. However, the frequency domain contains most of the information and features. The convolutional filters actually transform the signal from the time domain to the frequency domain. For this reason, the CNN model was selected, shown in Figure 17. For this experiment, a three-layer convolutional layer was used with Relu activation and batch-normalization in each layer. In this model, we used residual connection to concatenate all three convolutional layers to reduce overfitting.

The hyperparameters of the CNN model were selected empirically, which was based on simulation results. The simulation, included in our GitHub for reference, allowed us to test the measurements and effects of each potential hyperparameter. The following are the hyperparameter set from which the best values were selected (bold represents best values):

Batch_size = [16, **32**, 64, 512]
Window_size = [50, 100, 150, **200**, 250, 500]
Optimizer = [SGD, RMSprop, **Adam**, Adadelta, Adagrad, Adamax]
Learning rate = [**0.001**, 0.005, 0.01, 0.1]

After extracting the frequency domain features from the signal, the convoluted features were fed into dense neural network for classification. A three-dense-layer was used with Relu activation function. To reduce overfitting, a drop out was used in every dense layer.

Optimizer = Adam
Loss function = Categorical cross entropy
Batch size = 32
Epochs = 10

**Figure 18:**
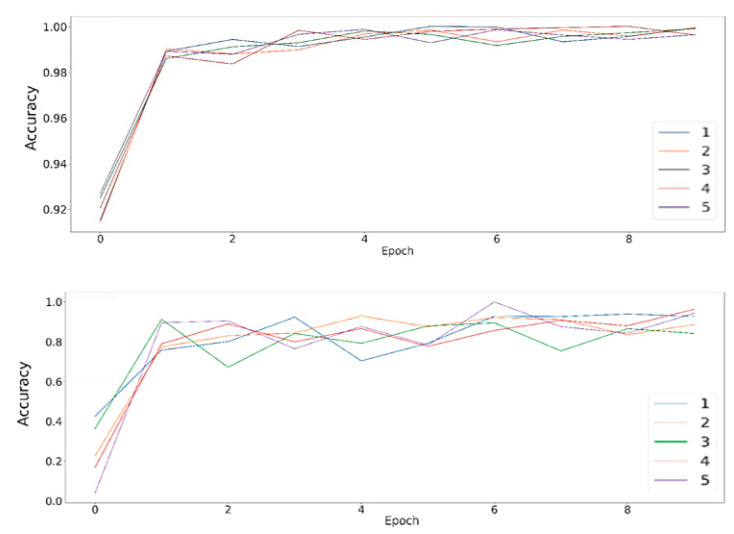
Training and validation accuracy.

**Figure 19:**
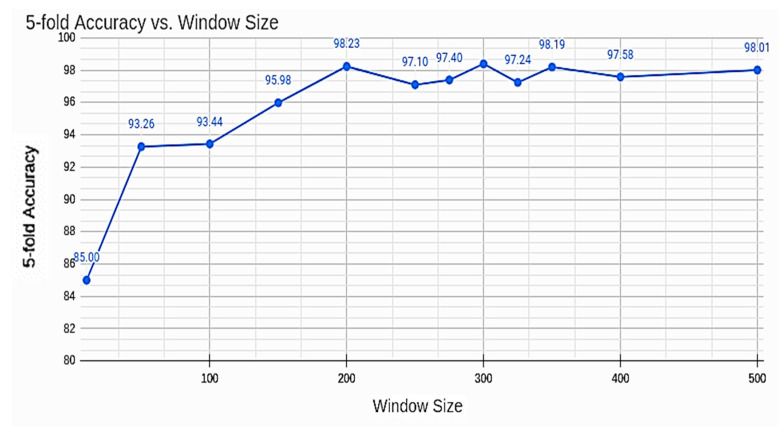
Overall k-Fold accuracy vs. windows size.

Overall, two important hyperparameters included tunes, which are batch-size and window-size for data preparation. Window size was most important, as it captured the vehicle specific movement. The window size was chosen from a range of 10–500 to determine the best one by comparing the 5-fold test accuracy. While the accuracy improvement over epochs can be seen in Figure 18, the best accuracy of 98.23% is demonstrated in Figure 19 at a window size of 200, and as the window size is increased from 200, the accuracy remains similar, with only 1% deviation, which represents that the unique pattern can be captured by using a window size of at least 200. A window size of more than 200 will also work, as it will also capture the pattern. 

The data were split into two categories randomly. To prepare the framework, 70% of the data were used, while the rest, the remaining 30%, was used to test it. The training data were subsequently split into two pieces again, at random. A total of 90% of the data were then used for training, and 10% was used for validation.

The five-fold cross validation and training accuracy is shown in Figure 18 below. Here, the model achieved an accuracy of 98% within only 10 epochs.

Figure 19 shows the 5-fold accuracy against window size, demonstrating the overall accuracy reaching 98% repeatedly, as the 5-fold accuracy was calculated with a window size of between 200–500.

### Alternative Setup Results

Furthermore, we also looked at the cross-weather, cross-device, and cross-vehicle performance. The accuracy degraded from 98% to 96% for cross-weather and 93% for cross device–vehicle, which was quite expected. The weather had a visible effect on driving patterns, which was the reason for recognition performance deviation of around 2%. However, the accuracy declined around 5% for cross-device and cross-vehicle since there might have been an impact of device change, adding to the impact of vehicle change.

**Cross weather (with data are collected by Samsung S8)**
Training set: Clear weather car
Test set: Rainy weather car
Accuracy: 96.32%
**Cross Device and Cross Vehicle (with data collected during clear weather)**
Training set: Honda Insight 2013 using Samsung S8
Test set: Toyota Camry 2005 using Pixel 4XL
Accuracy: 93.2%

## 5. Limitations

We selected our model carefully to fit our dataset, and compared our data across multiple machine learning models, as seen in Table 2, to arrive at our CNN model resulting as the most accurate model. Different machine learning models were trained to fit our dataset; however, they did not show high accuracy. Therefore, our vehicle dataset would not be easy to solve with most machine learning models. That said, a limitation of our study is that we did not run a separate quality dataset of another high-performing study that focused on accelerometers and/or gyroscopes through our own model [26]. Doing this would further confirm the validity of our results against other best practices in vehicle recognition with smart sensor research.

In addition, we did not implement our own cost-effectiveness study, but relied on previous literature. This is a limitation that future research can address to find the specific cost-effectiveness of using our two sensors, as opposed to three or more. Moreover, although we tested one specific mount (Vicseed universal dashboard cellular mount), and also tested results with the smartphone lying on a seat, it is highly possible that other mounts could possibly affect the outcomes of the study. For example, the forced air hitting a smartphone mounted to an automobile’s heating or air conditioning vent would potentially cause vibrations in the cell phone that could alter its gyroscope or accelerometer readings. This is a limitation of our study, and further research should be done to thoroughly examine the effect of different mounts on smart device sensor vehicle recognition.

## 6. Discussion and Future Research

This study’s model was consistent with our research question and desired outcomes. Repeatedly, the results achieved an overall accuracy of 98% within 10 epochs, when 5-fold accuracy was calculated with a window size of between 200–500. This overall accuracy applied to the car, bus, rail, or bicycle recognition, and was not affected by weather or traffic conditions when testing the car. Moreover, the make of the car (Honda or Toyota) also did not affect accurate recognition of the car.

In achieving these results, the study advances a branch of vehicle identification that can passively acquire vehicle and activity data via smart-device sensors. With the ability to recognize a vehicle through smart devices, this paper also contributes to a growing body of research on sensor-based systems that can track the transportation habits of drivers and passengers, potentially leading to systems that can understand and even anticipate the preferred mode of transportation of the passengers and drivers. Furthermore, based on parameters such as distance and time travelled, conditions of the road, and other driving conditions, the optimal mode of transportation could also be determined and recommended. These conclusions are consistent with the evidence and arguments presented in our manuscript. Our model was able to consistently identify the mode of transportation for bus, bicycle, rail, and automobile. Moreover, this identification was consistent regardless of make of the car, weather conditions, traffic conditions, or whether the car was a hybrid or standard gasoline vehicle.

The lives of many people have been saved all over the world as a result of GPS technologies, which have allowed for automatic wire-free vehicle tracking. These systems not only warn users of an accident, but they also assist drivers in preventing collisions. GPS devices and motion detection technologies can be used to monitor the progress of vehicles. However, GPS technology has the downside of being unable to track small movements. Nonetheless, by using an accelerometer and gyroscope, we are able to recognize small vehicle displacement with ease, and classify the transportation mode. With this ability to track small-scale vehicle movements, vehicles can be classified and monitored in a way that is similar, but offers different benefits and also drawbacks, in comparison to not only GPS, but also other intelligent transportation systems.

Via smart-device sensor vehicle recognition, it is possible that vision-based surveillance systems can benefit from a growing body of IoT technology, rapidly becoming embedded in vehicles. With this in mind, there are numerous potential benefits of vehicle recognition via smart-device sensors surface, including extra features of safety that employ wireless technology to make a direct communication with or track motor vehicles’ movement. This is because the mobile-type systems inside vehicles can include the same smart watch sensors that we utilized in our vehicle recognition research, and, in doing so, can enable a synchronicity between what is happening inside the vehicle with what occurs through external surveillance and monitoring. This simultaneous use of vehicle recognition systems would, in future research, allow for the synchronization of external and internal imaging, allowing the potential for insurance, policing, and vehicle monitoring systems to know, potentially in real time, how the behaviors of the vehicle, the behaviors of the driver, and the environment around the vehicle (such as traffic, weather, and other vehicles) combine to cause traffic, vehicular accidents, and potentially to both better prevent and understand incidents as they happen on the road.

Hence, vehicle recognition via smart-device sensors may address many functional applications and theoretical directions within the present body of literature. As shown in Table 1, numerous important studies that have advanced theory in vehicle recognition and have led to practical real-world applications may benefit through an exploration of vehicle recognition via smart-device sensors research. Nevertheless, it is important to also point out that, while smart sensors have the potential to add to the sophistication and utility of vehicle recognition systems, there are potential risks of smart phone use while driving. According to a recent report, mobile phones (especially texting) are involved in no less than 23% of all car crashes, in addition to a total of 1.3 million United States accidents [86]. In that research study, a smartphone was used as a sensor to detect the simultaneous behavior of driving and texting, which is similar to our paper, where we use a smartphone as a sensor in order to obtain data. The researchers further proposed TEXIVE, which can perfectly recognize hazardous activities with high sensitivity, precision, and accuracy by putting in leverage the inertial sensors merged with regular smartphones. Extensive experiments entailing a variety of individuals who volunteered on various smartphones and vehicles were conducted, and the outcomes indicated that TEXIVE had an accuracy of 87.18% in terms of classification, and a precision rate of 96.67%. Studies such as this highlight that any smart sensor technology meant for use while driving must be one that does not require the intrusive or distracting use of mobile phones to conflict with attentiveness of the vehicle operator.

## Figures and Tables

**Table 1:** Vehicle Recognition Sensor Types and Definitions with Relevant Studies.

Sensor	Technical Function	Articles Using Sensor for Vehicle Recognition
Used only accelerometer and/or gyroscope	Equal or more cost-effective than our study, utilizing just accelerometers and/or gyroscopes	Pias et al., 2020 [4]; Suharjono et al., 2019 [33]; Kaewunruen et al., 2021 [26]; Fang et al., 2016 [35]; Sengül et al., 2021 [34]
Accelerometer	Measures linear acceleration, directional movement, and three-dimensional object orientation or stationing, as well as changes in the ambient environment	Xia et al., 2014 [3]; Alotaibi, 2020 [18]; Badii et al., 2021 [20]; Gjoreski et al., 2021 [21]; Erdelic et al., 2022 [23]; Guvensan et al. 2017 [25], Iskanderov and Guvensan, 2020 [13]; Jeyakumar et al., 2018 [6]; Kaewunruen et al., 2021 [26]; Martin et al., 2017 [10]; Şengül et al., 2021 [34]; Shafique and Hato, 2020 [11]; Tregel et al., 2018 [12]; Wang et al., 2018 [40]; Wang et al., 2021 [8]; Xiao et al., 2019 [41]; Lu et al., 2018 [30]; Suharjono et al., 2019 [33]; Thomas et al., 2018 [37]; Qin et al., 2018 [31]
Gyroscope	Measures vibrations in any direction	Alotaibi, 2020 [18]; Gjoreski et al., 2021 [21]; Balli and Sağbaş, 2017 [22]; Erdelic et al., 2022 [23]; Guvensan et al. 2017 [25]; Iskanderov and Guvensan, 2020 [13]; Jeyakumar et al., 2018 [6]; Şengül et al., 2021 [34]; Wang et al., 2018 [40]; Wang et al., 2021 [8]; Qin et al., 2018 [31]; Lu et al., 2018 [30]
GPS	Contains user geographic location and timestamp	Xia et al., 2014 [3]; Badii et al., 2021 [20]; Balabka and Shkliarenko., 2021 [14]; Bjerre-Nielsen et al., 2020 [16]; Dogan et al., 2021 [5]; Iabanzhi et al., 2021 [7]; Li et al., 2021 [28]; Martin et al., 2017 [10]; Ren 2021 [32]; Tregel et al., 2018 [12]; Tian et al., 2021 [38], Thomas et al., 2018 [37]; Xiao et al., 2019 [41]; Zhu et al., 2021 [42]; Shafique and Hato, 2020 [11]
Magnetometer	Measures and processes magnetic signals as a result of changes in the ambient magnetic field	González et al., 2020 [17]; Gjoreski et al., 2021 [21]; Balli and Sağbaş, 2017 [22]; Erdelic et al., 2022 [23]; Guvensan et al. 2017 [13]; Iskanderov and Guvensan, 2020 [13]; Jeyakumar et al., 2018 [6]; Lou et al., 2018 [29]; Wang et al., 2018 [40]; Wang et al., 2021 [8]; Lu et al., 2018 [30]; Lan et al., 2010 [27]; Qin et al., 2018 [31],
Wi-Fi sensor	Contains the user’s identification and wireless fidelity signal strength	Bjerre-Nielsen et al., 2020 [16]; Dogan et al., 2021 [5]; Iabanzhi et al., 2021 [7]; Li et al., 2021 [28]; Tian et al., 2021 [38]; Thomas et al., 2018 [37]; Ren 2021 [32]; Xiao et al., 2019 [41], Zhu et al., 2021 [42].
Gravity sensors	Measures gravitational force	Erdelic et al., 2022 [23], Jeyakumar et al., 2018 [6]
Barometer	Measures ambient and inertial pressure.	Wang et al., 2018 [40]; Qin et al., 2018 [31]
Bluetooth sensor	Contains user identification, timestamp, and signal strength	Bjerre-Nielsen et al., 2020 [16]

**Table 2:** Accuracy of traditional ML classifiers and CNN.

Classifier	Accuracy
Logistic Regression	45.36%
Naive Bayes	58.13%
SVM	76.12%
XGBoost	84.73%
**CNN**	**98%**

## Data Availability

Data for this study is available at: https://github.com/PiasTanmoy/Vehicle-Recognition-Using-Smart-Sensors (accessed on 5 June 2022).
